# Wet-Bulb Globe Temperature, Universal Thermal Climate Index, and Other Heat Metrics for US Counties, 2000–2020

**DOI:** 10.1038/s41597-022-01405-3

**Published:** 2022-06-17

**Authors:** Keith R. Spangler, Shixin Liang, Gregory A. Wellenius

**Affiliations:** 1 Boston University School of Public Health, Department of Environmental Health, Boston, MA USA; 2 Boston University, Department of Mathematics & Statistics, Boston, MA USA

**Keywords:** Climate sciences, Environmental health

## Abstract

Epidemiologic research on extreme heat consistently finds significant impacts on human morbidity and mortality. However, most of these analyses do not use spatially explicit measures of heat (typically assessing exposures at major cities using the nearest weather station), and they frequently consider only ambient temperature or heat index. The field is moving toward more expansive analyses that use spatially resolved gridded meteorological datasets and alternative assessments of heat, such as wet-bulb globe temperature (WBGT) and universal thermal climate index (UTCI), both of which require technical geoscientific skills that may be inaccessible to many public health researchers. To facilitate research in this domain, we created a database of population-weighted, spatially explicit daily heat metrics – including WBGT, UTCI, heat index, dewpoint temperature, net effective temperature, and humidex – for counties in the conterminous United States derived from the ERA5-Land gridded data set and using previously validated equations and algorithms. We also provide an R package to calculate these metrics, including gold-standard algorithms for estimating WBGT and UTCI, to facilitate replication.

## Abstract (summary)

**Table:** 

Measurement(s)	ambient temperature • dewpoint temperature • humidex • heat index • wet-bulb globe temperature • universal thermal climate index • effective temperature
Technology Type(s)	reanalysis
Sample Characteristic - Environment	2-meter atmospheric conditions
Sample Characteristic - Location	contiguous United States of America

## Background & Summary

Exposure to moderate and extreme heat is associated with increased risk of illness and death^1^. Although most epidemiologic studies contributing to this conclusion use dry-bulb temperature (i.e., ambient air temperature measured in the shade) as the exposure metric of interest^2–6^, others have assessed alternative metrics that incorporate humidity – such as the heat index, a combined temperature and humidity metric used by the U.S. National Weather Service^7^, or the humidex, which is used by the Meteorological Service of Canada – to better estimate the physiologic impact of heat on the human thermoregulatory system^8–10^. Increasingly, public health research on heat is considering metrics that combine additional meteorological conditions, including solar radiation and wind speed, to further contextualize the actual heat stress experienced by populations^11–13^.

Of particular interest is the wet-bulb globe temperature (WBGT), a thermal index originally developed in the 1950s to establish epidemiologically relevant thermal thresholds to prevent heat-related illnesses at US military training camps^14^. The WBGT is a weighted average of the ambient, wet-bulb, and globe temperatures, which collectively incorporate thermal, solar, and convective heat transfers from ambient temperature, humidity, solar radiation, and wind speed^15^. In contrast to simpler, more commonly used metrics, such as ambient temperature or heat index, WBGT is measured in conditions of direct solar radiation and is partially mitigated by wind speed, making it an appealing measure for estimating thermal conditions experienced by outdoor workers and athletes. In recognition of this utility, it has been approved by the International Organization for Standardization (ISO), the American Conference of Governmental Industrial Hygienists, and other national and international organizations for use as a thermal stress screening tool^16^.

However, it should be noted that WBGT has limitations, including its potential underestimation of thermal stress in conditions where sweating is restricted, susceptibility to measurement errors, and variability based on clothing and activity^14^. Others have also noted that the scale of the measurement is prone to misinterpretation, given that extreme values of WBGT are much lower than what would be considered extreme by ambient temperature standards^16^. Newer metrics have been developed that may reduce some of these limitations; of interest here is the Universal Thermal Climate Index (UTCI), a heat metric derived from human energy balance models with the goal of being universally applicable, physiologically relevant, and appropriate for use in a range of bioclimatic applications^17^. Although the UTCI is correlated with WBGT and may similarly reflect thermal perception^18^, some have advocated for replacing WBGT with UTCI in operational use cases, particularly in athletics^19,20^.

While WBGT and UTCI have clear utility in bioclimatic contexts, there are challenges for population health researchers hoping to use them in their analyses. For example, measuring WBGT requires specialized equipment that is not widely deployed operationally, and UTCI similarly requires data that are frequently unavailable in meteorological data sets. Although it would be advantageous for public health researchers to have easy access to population-scale estimates of WBGT, UTCI, and other heat metrics, no such database presently exists. Furthermore, no single measure of heat will be universally superior in all contexts^10,19^, suggesting the need for a single dataset containing multiple metrics for intercomparison.

Concurrently, heat-health researchers are increasingly aware of the value in using gridded meteorological data sets, both for their potential to provide population-applicable estimates of weather experienced across large areas and to avail rural areas without weather stations to epidemiologic analyses^21^. Recent studies have demonstrated the utility of a range of gridded data sets for such applications^22–25^. Although these data sets are a valuable source of information, they contain massive amounts of data that require time, computational resources, and expertise to process that are not available to many public health researchers.

Given these barriers to enhanced epidemiologic research, the broader goal of this data set is to provide: (1) a data set of pre-processed gridded data, population-weighted to the daily county level; and (2) code for calculating various heat metrics, including wet-bulb globe temperature and the Universal Thermal Climate Index using gold-standard approaches. The potential reuse value of these data extends not only to epidemiologic analyses, but to any research that requires daily, county-level estimates of temperature or other heat metrics.

## Methods

### Overview

**Fig. 1:**
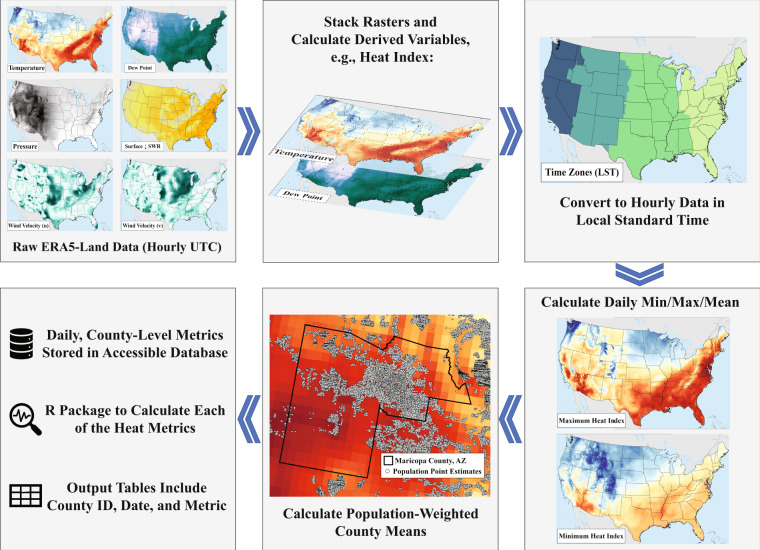
Schematic overview of the creation of the *heatmetrics* database.

Our dataset provides daily minimum, maximum, and mean values of ambient temperature, dew-point temperature, net effective temperature, heat index, humidex, wet-bulb globe temperature, and the Universal Thermal Climate Index, all population-weighted at the county level. We used a high-resolution reanalysis dataset to calculate these hourly heat metrics across the entire contiguous United States (CONUS) from January 2000 to December 2020. We developed an accompanying R package (*heatmetrics*) that can be used to replicate the calculations on other data sets (Fig. 1).

### Reanalysis data

We derived a series of heat metrics using data from the European Centre for Medium-Range Weather Forecasts (ECMWF) Reanalysis v5 Land product (ERA5-Land)^26,27^. The ERA5-Land variables we obtained included: (1) two-meter air temperature, (2) two-meter dew point temperature, (3) surface pressure, (4) ten-meter zonal and meridional wind velocity vectors, (5) surface solar radiation downward, (6) surface thermal radiation downward, (7) surface net solar radiation, and (8) surface net thermal radiation. The ERA5-Land data are available hourly at a spatial resolution of 0.1 degrees (approximately 9 km) globally over land. We also obtained total sky direct solar radiation at surface from ERA5^28,29^, the reanalysis from which ERA5-Land is derived. We interpolated this from the 0.25-degree ERA5 grid to the 0.1-degree ERA5-Land grid using nearest-neighbor interpolation, following the approach of Yan *et al*.^30^.

### Converting to local standard time

Data from ERA5-Land are provided hourly in Coordinated Universal Time (UTC), also known as Greenwich Mean Time (GMT). Since many users need minimum and maximum values relative to the *local* day, we created raster stacks of the calculated hourly data that aligned with the local time zone. To do this, we created rasters of ERA5-Land grids containing centroids of latitude and longitude and then used the *lutz* R package^31^ to identify the time zone of each grid cell, which was represented as a numeric offset from UTC time. We created separate rasters for standard time and daylight saving time (for example, Eastern Standard Time has a UTC offset of −5 hours while Eastern Daylight Time has an offset of −4 hours). While most locations had consistent time zones throughout the study period, we manually accounted for time zone changes that occurred in the state of Indiana in 2006 and 2007 by creating shapefiles of affected counties and adjusting the ERA5-Land grid offsets accordingly. The final result was eight rasters representing grid-level UTC offsets: four each for local standard time (LST) and local daylight time (LDT) for years 2000–2005, 2006, 2007, and 2008–2020. We used these time zone rasters as masks to subset the calculated heat metrics corresponding to the local day. The result was stacks of 24 hourly observations for each day that reflected data from 00 local time to 23 local time. We note that although hourly time steps are the highest temporal resolution available in reanalysis data, “true” daily minimum and maximum temperatures can occur between hours. Others have found that, in some instances, this can lead to small differences in estimated relative risks of exposure to ambient heat, but that such differences have considerably smaller magnitudes than the differences in relative risks found between different heat metrics^32^.

### Calculating heat metrics

We calculated hourly heat metrics across all of CONUS between January 2000 and December 2020 on the stacks of ERA5-Land data converted to local time. We then calculated, at the pixel level, the minimum, maximum, and mean values for each variable on every local day. The final results were rasters of CONUS for daily observations of each of the heat metrics described in this section. The database may be updated in the future as ERA5-Land data are updated.

#### Wet-bulb globe temperature

Lemke and Kjellstrom^33^ summarized and compared approaches to estimating wet-bulb globe temperature, and they found that the algorithm by Liljegren *et al*.^15^ performed most accurately across a range of conditions. We therefore employed this algorithm in our calculations here. In contrast to most estimates of WBGT – which use relatively simple equations – the Liljegren approach applies fundamental laws of physics, thermodynamics, and mass balance to separately model natural wet bulb (T_W_) and globe temperatures (T_G_), which together comprise 90% of the WBGT (the remaining 10% is the ambient temperature [T_A_]; Eq. 1).

1 $$WBGT=0.70\cdot {T}_{W}+0.20\cdot {T}_{G}+0.1\cdot {T}_{A}$$

The Liljegren algorithm was originally written in the C programming language but was translated to Javascript and made available in a web interface by the Occupational Safety and Health Administration (OSHA)^34^. We translated this Javascript into an R package and processed it on raster stacks of the ERA5-Land data.

The WBGT algorithm requires as inputs the following variables (Table 1): year, month, day, hour, minute, UTC offset, averaging time of meteorological observations, latitude, longitude, temperature, relative humidity, incident solar radiation, wind speed, surface pressure, height of wind speed measurement, vertical temperature difference between observed temperature and wind-speed-height temperature, and urban or rural land cover. Although most of these variables are provided directly by ERA5-Land, a few needed to be derived first. Relative humidity was calculated using air temperature and dew-point temperature, and the wind speed was calculated using the zonal and meridional wind velocity vectors (Table 1). To identify ERA5-Land grid cells as urban or rural, we used the 2011 National Land Cover Database^35^ and assigned grid cells as “urban” (1) if at least 33% of the 30-meter by 30-meter land cover pixels within each ERA5-Land grid cell were classified as “developed, low intensity,” “developed, medium intensity,” or “developed, high intensity.” All other grid cells were classified as “rural” (0). Note that the contribution of urban/rural to the WBGT calculation is marginal and only affects the conversion of wind speed from higher altitudes to two-meter equivalents.

**Table 1:** Inputs to wet-bulb globe temperature (WBGT) algorithm.

Variable	Calculation	Units/Notes
Year	Provided by ERA5-Land	Numeric (YYYY)
Month	Provided by ERA5-Land	Numeric (MM), 1–12
Day	Provided by ERA5-Land	Numeric (DD), 1–31
Hour	Provided by ERA5-Land	Numeric (HH), 0–23 in local time
Minute	Set to 0 for all observations	Top-of-hour observations
UTC Offset	Set to 0 for all observations because we calculated on the original UTC data	Number of hours difference from UTC, if applicable
Averaging Time	Set to 0 for all observations	Observations are provided as either instantaneous or already averaged over the hour
Latitude	ERA5-Land grid centroids	Decimal-degrees North (°N)
Longitude	ERA5-Land grid centroids	Decimal-degrees East (°E)
Ambient Temperature	Provided by ERA5-Land	Degrees Celsius (°C)
Relative Humidity	$e=610.94\cdot exp\left(\frac{17.625\cdot {T}_{D}}{243.04+{T}_{D}}\right)$	T_D_ = dew point temperature (°C); see Lawrence (2005)^49^
	${e}_{s}=610.94\cdot exp\left(\frac{17.625\cdot {T}_{A}}{243.04+{T}_{A}}\right)$	T_A_ = ambient air temperature (°C); see Lawrence (2005)^49^
	$RH=100\cdot \left[\frac{e}{{e}_{s}}\right]$	e = vapor pressure (Pascals [Pa]) and e_s_ = saturation vapor pressure (Pascals [Pa])
Surface Solar Radiation Downward	Provided by ERA5-Land	Watts per square-meter (W/m^2^)
Wind Speed	$ws=\sqrt{{u}^{2}+{v}^{2}}$	u = zonal wind velocity (m/s) and v = meridional wind velocity (m/s)
Surface Pressure	Provided by ERA5-Land	Pascals (Pa)
Wind-Speed Height	Set to 10 meters for all observations	N/A
Temperature Lapse	Set to −0.052 °C for all observations	Assumes vertical lapse rate of −6.5 °C / km
Urban	Assigned 1 (urban) to pixels classified as urban or built up in the National Land Cover Database 2011 dataset and 0 (rural) otherwise	N/A

In addition to the pre-processing described above, we made a few small modifications to the Liljegren algorithm, as described here and as comments throughout the R source code. First, in the calculation of the natural wet-bulb temperature, the original algorithm used a static enhancement factor of 1.004 when calculating the saturation vapor pressure; however, this factor assumes a barometric pressure of at least 800 hPa. To accommodate lower pressures for high-elevation locations, we applied an enhancement factor that is a function of barometric pressure (second quantity of Eq. 2), following Equation 8 in Buck (1981, p. 1532)^36^.

2 $${e}_{sat}=\left(6.1121\cdot \exp \left[\frac{17.502\cdot \left({T}_{air}-273.15\right)}{{T}_{air}-32.18}\right]\right)\cdot \left(1.0007\cdot 3.46\cdot 1{0}^{-6}\cdot {P}_{air}\right)$$

Where T_air_ is the ambient temperature in Kelvin and P_air_ is the barometric pressure in hPa.

Second, we fixed a small error in the stability classes, which are used to estimate the 2-meter wind speeds when the input wind speeds are measured at a different height (as is the case in ERA5-Land, which reports 10-meter wind speeds). In the original algorithm, nighttime conditions with wind speeds between 2 and 2.5 m/s were given stability classes of “E” and “F” for positive and negative lapse rates, respectively; we changed this to the correct values of “D” and “E,” consistent with guidance from EPA documentation (see Table 6–7 in reference)^37^.

Third, we updated the algorithm for calculating the heat of vaporization to follow the approach of Meyra *et al*.^38^, which was found to be more accurate than the traditional Watson equation. Although we believe this to be a more-accurate method, we find that it typically changes the estimate of the wet-bulb temperature by less than 0.1 degrees Celsius.

Finally, we changed the minimum wind speed from 0.13 m/s to 0.5 m/s for a more-conservative estimate of WBGT that prevents unreasonably high estimates that result from very low wind speeds. Lemke and Kjellstrom (2012)^33^ recommend a more-conservative minimum wind speed of 1 m/s for assessing WBGT effects on outdoor workers, noting that typical bodily movement results in an apparent wind speed on the skin of at least 1 m/s. Our value of 0.5 m/s is a balance between these values and helps capture the WBGT for stationary individuals, as our index is not exclusively for outdoor workers. Note that this wind speed adjustment was done as a pre-processing step and that the minimum wind speed is only directly set to 0.5 m/s in the WBGT function of the *heatmetrics* R package when supplying wind speeds at a height other than two meters.

We conducted a sensitivity analysis to determine the impact that these changes to the Liljegren algorithm had on the final county-level mean values. We found that daily maximum WBGT estimates for August 2020 across all available CONUS counties using WBGT algorithms with and without the aforementioned alterations were extremely similar, with *r*^2^ = 99.99%, a mean absolute difference of 0.08 °C, and a maximum absolute difference of 0.25 °C.

#### Universal Thermal Climate Index (UTCI)

We calculated UTCI following the approach of Di Napoli *et al*.^39^, as implemented in the ECMWF python library, *thermofeel*^40^. This method uses the sixth-order polynomial regression approximation given by Bröde *et al*. (2011), which is a highly accurate approximation of UTCI with a root-mean square error of 1.1 degrees Celsius^41^. The equation takes as inputs the ambient temperature, 10-meter wind speed, vapor pressure, and mean radiant temperature (T_mrt_). We calculated mean radiant temperature following the approach of Di Napoli *et al*.^42^, which approximates T_mrt_ using hourly measurements of total downward surface solar radiation (direct and diffuse), surface *net* solar radiation, downward surface thermal radiation, surface *net* thermal radiation, downward direct surface solar radiation, and cosine of the solar zenith angle (cza). As was done in Di Napoli *et al*.^42^, we calculated the average *daytime* cza in order to minimize errors that arise during sunrise and sunset hours, described comprehensively by Hogan and Hirahara^43^. We used this same integrated cza approach in the calculation of WBGT and note that, although it is the most-accurate approach, others have found that it actually has a very small impact (<0.01 °C on average) on the overall estimation of UTCI^40^. Although the Bröde *et al*. (2011) algorithm is suitable for wind speeds up to 30.3 m/s, we followed Di Napoli *et al*. (2021) in capping wind speeds at 17 m/s and marking as missing (“NA”) observations above this threshold, based on findings of extremely low UTCI values at these tropical-storm-force wind speeds^44^. Finally, consistent with recommendations in Bröde *et al*.^41^, we constrained the vapor pressure input to be consistent with a relative humidity of ≥5%, the lower bound for which their algorithm is validated: in cases where relative humidity was less than 5%, we set the vapor pressure equal to the saturation vapor pressure multiplied by 0.05. This had only a minimal impact on the final county UTCI values: 99.93% of county-day maximum UTCI values were completely unaffected. Among the county-days that did have the adjustment, the mean absolute difference in maximum UTCI was 0.06 °C and the maximum absolute difference was 0.58 °C.

#### Other heat metrics

Most of the other heat metrics in this data set use relatively straightforward equations (Table 2). The one exception is heat index: we calculated this variable using the *weathermetrics* R package^7^, which implements the approach to calculating heat index that is used by the National Weather Service. Note that, for consistency with how we calculated WBGT, we set the minimum wind speed to 0.5 m/s for all variable calculations that use wind speed.

**Table 2:** Summary of the methods used to calculate the heat metrics in the data set.

Variable	Calculation	Variables/Units	Ref.
Ambient Temperature	Provided directly by ERA5-Land	°C	N/A
Dew-Point Temperature	Provided directly by ERA5-Land	°C	N/A
Net Effective Temperature	$37-\left(\frac{37-{T}_{A}}{0.68-0.0014\cdot RH+\frac{1}{1.76+1.4\cdot {w}^{0.75}}}\right)-\left[0.29\cdot {T}_{A}\cdot \left(1-\frac{RH}{100}\right)\right]$	RH – rel. hum. (%) T_A_ – air temp. (°C) w – wind speed (m/s)	^50^
Humidex	$e=6.1094\cdot exp\left(\frac{17.625\cdot {T}_{D}}{243.04+{T}_{D}}\right)$	T_D_ – dew-point T (°C)	^49^
	${T}_{A}+\frac{5}{9}\cdot \left[e-10\right]$	T_A_ – air temp. (°C) e – vapor pressure (hPa)	^51^
Heat Index	*Weathermetrics* R package following algorithm of National Weather Service, using ambient temperature and dew point temperature as inputs	°C	^7^
Wet-Bulb Globe Temperature (WBGT)	Liljegren *et al*. approach as described in the Methods section here and in the reference.	°C	^15^
Universal Thermal Climate Index (UTCI)	Mean radiant temperature (T_mrt_) estimated using Di Napoli *et al*. (2020) approach	°C	^42^
	Bröde *et al*. (2011) approach as implemented by Di Napoli *et al*. (2021) and Brimicombe *et al*. (2022). See Methods for details.	°C	^39–41^

### Calculating population-weighted county means

Daily heat metric values are reported as population-weighted county mean values. We used high-resolution (approximately 250 m × 250 m) population data from the Joint Research Centre (JRC) of the European Commission^45^ to calculate spatial weights for each ERA5-Land grid cell within each county based on the proportion of the county population residing in that grid cell. For example, if the sum of the high-resolution population points within a particular grid cell in County A were equal to 10% of the total county population, then that grid cell would get weighted 10% toward the overall county mean. Temperatures in the more densely populated parts of counties were thus weighted more heavily than the less-populous parts, resulting in metrics that are likely more relevant to population-based studies. To account for potential population shifts over the period analyzed, we used two sets of population estimates based on availability in the JRC data: we used 2000 population distributions for county means from 2000–2009 and 2015 populations for 2010–2020.

To account for missing data, we added flag variables to indicate whether the county estimates for a particular day were based on non-missing grid cells representing less than 50% of the population. This is applicable because ERA5-Land is available only over land areas in which the grid cell is comprised of no more than 50% ocean; this ensures that the meteorological data are representative of land areas, but it also means that some small island and coastal areas are excluded. An additional source of missing data is from particular hourly values being marked as NA (for example, hourly UTCI values when wind speeds exceed 17 m/s). A grid cell was marked as NA for a particular variable-day if fewer than 21 hourly observations were available. We calculated the percent of county populations that were represented by non-missing ERA5-Land data for every variable on every day and added flags as follows: “0” means the estimate is based on data representing ≥50% of the population, “1” covers 10–49% of the population, “2” covers <10%, and “3” means the data are completely missing (variable-days in this case are marked as NA). These flags affect only an extremely small portion of the data set: >99.7% of county-days have no flag, and only two counties in all of CONUS are missing entirely from the data set (Monroe County, Florida [containing the Key West archipelago] and Nantucket County, Massachusetts [containing the island of Nantucket]).

## Data Records

The *heatmetrics* data are accessible via figshare^46^. The variables currently available for download are described in Table 3. At present, the data set includes population-weighted estimates at the county level, which can be queried using the state-county Federal Information Processing Standard (FIPS) identifier. We also provide a separate data set of *unweighted* county mean values, which were created by taking a simple average of all grid cells in a county, which are also available via figshare^47^. Variable names are the same between the two data sets, so users should take care to download the applicable file for their needs and rename variables as appropriate if using both data sets simultaneously.

**Table 3:** Description of variables available in the heatmetrics database.

Variable Name (Short)	Variable Name (Long)	Description / Format	Units
StCoFIPS	State-county Federal Information Processing Standard (FIPS) Identifier	Unique county identifier: concatenation of two-digit state identifier and three-digit county identifier	N/A
Date	Date	Local day in the format YYYYMMDD	N/A
Tmin_C	Daily Minimum Ambient Temperature	Lowest 2-meter ambient temperature observed from hourly data from 00 LST to 23 LST	°C
Tmax_C	Daily Maximum Ambient Temperature	Highest 2-meter ambient temperature observed from hourly data from 00 LST to 23 LST	°C
Tmean_C	Daily Mean Ambient Temperature	2-meter ambient temperature averaged over hourly observations from 00 LST to 23 LST	°C
TDmin_C	Daily Minimum Dew Point Temperature	Lowest dew point temperature observed from hourly data from 00 LST to 23 LST	°C
TDmax_C	Daily Maximum Dew Point Temperature	Highest dew point temperature observed from hourly data from 00 LST to 23 LST	°C
TDmean_C	Daily Mean Dew Point Temperature	Dew point temperature averaged over hourly observations from 00 LST to 23 LST	°C
NETmin_C	Daily Minimum Net Effective Temperature	Lowest net effective temperature observed from hourly data from 00 LST to 23 LST	°C
NETmax_C	Daily Maximum Net Effective Temperature	Highest net effective temperature observed from hourly data from 00 LST to 23 LST	°C
NETmean_C	Daily Mean Net Effective Temperature	Net effective temperature averaged over hourly observations from 00 LST to 23 LST	°C
HImin_C	Daily Minimum Heat Index	Lowest heat index observed from hourly data from 00 LST to 23 LST	°C
HImax_C	Daily Maximum Heat Index	Highest heat index observed from hourly data from 00 LST to 23 LST	°C
HImean_C	Daily Mean Heat Index	Heat index averaged over hourly observations from 00 LST to 23 LST	°C
HXmin_C	Daily Minimum Humidex	Lowest humidex observed from hourly data from 00 LST to 23 LST	°C
HXmax_C	Daily Maximum Humidex	Highest humidex observed from hourly data from 00 LST to 23 LST	°C
HXmean_C	Daily Mean Humidex	Humidex averaged over hourly observations from 00 LST to 23 LST	°C
WBGTmin_C	Daily Minimum Wet-Bulb Globe Temperature	Lowest wet-bulb globe temperature (WBGT) observed from hourly data from 00 LST to 23 LST	°C
WBGTmax_C	Daily Maximum Wet-Bulb Globe Temperature	Highest wet-bulb globe temperature (WBGT) observed from hourly data from 00 LST to 23 LST	°C
WBGTmean_C	Daily Mean Wet-Bulb Globe Temperature	Wet-bulb globe temperature (WBGT) averaged over hourly observations from 00 LST to 23 LST	°C
UTCImin_C	Daily Minimum Universal Thermal Climate Index	Lowest Universal Thermal Climate Index (UTCI) observed from hourly data from 00 LST to 23 LST	°C
UTCImax_C	Daily Maximum Universal Thermal Climate Index	Highest Universal Thermal Climate Index (UTCI) observed from hourly data from 00 LST to 23 LST	°C
UTCImean_C	Daily Mean Universal Thermal Climate Index	Universal Thermal Climate Index (UTCI) averaged over hourly data from 00 LST to 23 LST	°C
Flag_T	Ambient temperature flag	Indicator of the percent of county population represented by the county-day ambient temperature estimate. 0: ≥50%, 1: 10–49%, 2: <10%, 3: 0% (NA)	N/A
Flag_TD	Dew point temperature flag	Indicator of the percent of county population represented by the county-day dew point temperature estimate. 0: ≥50%, 1: 10–49%, 2: <10%, 3: 0% (NA)	N/A
Flag_NET	Net effective temperature flag	Indicator of the percent of county population represented by the county-day net effective temperature estimate. 0: ≥50%, 1: 10–49%, 2: <10%, 3: 0% (NA)	N/A
Flag_HI	Heat index flag	Indicator of the percent of county population represented by the county-day heat index estimate. 0: ≥50%, 1: 10–49%, 2: <10%, 3: 0% (NA)	N/A
Flag_HX	Humidex flag	Indicator of the percent of county population represented by the county-day humidex estimate. 0: ≥50%, 1: 10–49%, 2: <10%, 3: 0% (NA)	N/A
Flag_WBGT	Wet-bulb globe temperature flag	Indicator of the percent of county population represented by the county-day WBGT estimate. 0: ≥50%, 1: 10–49%, 2: <10%, 3: 0% (NA)	N/A
Flag_UTCI	Universal Thermal Climate Index flag	Indicator of the percent of county population represented by the county-day UTCI estimate. 0: ≥50%, 1: 10–49%, 2: <10%, 3: 0% (NA)	N/A

## Technical Validation

The *heatmetrics* data set employs existing algorithms and an established reanalysis product that have all been peer-reviewed and frequently cited in the literature. Please see accompanying references and citations therein for the input data set used, ERA5-Land, for model development and validation^27^. The WBGT algorithm used here is based on the Liljegren approach, which was found to be accurate to within 1 °C in the developers’ testing^15^, and independently verified as being the most accurate across different estimation methods^33^. Similarly, we followed the approach of Di Napoli *et al.*^39^, as implemented by Brimicombe *et al*.^40^ for operational distribution through the European Centre for Medium-Range Weather Forecasts (ECMWF); this algorithm employs the UTCI approximation reported by Bröde *et al*. (2011), which was found to have a root-mean square error of approximately 1.1 °C.

### Disclaimers

This data set contains modified Copernicus Climate Change Service information (2022), as described and cited in the manuscript. Neither the European Commission nor ECMWF is responsible for any use that may be made of the Copernicus information or data it contains. The data set and software are provided by the manuscript authors “as is” with no warranty of any kind.

## Data Availability

We developed the *heatmetrics* R package to facilitate replication of these methods to other meteorological data sets. The package is available to download via figshare^48^.
